# Supplementary material for: An Ultra-Sensitive Multi-Functional Optical Micro/Nanofiber Based on Stretchable Encapsulation
Source: Sensors (Basel). 2021 Nov 9;21(22):7437. doi: 10.3390/s21227437 (PMC8618424; doi:10.3390/s21227437)
Supplement: Supplementary file 1 [file sensors-21-07437-s001.zip › sensors-1430837-supplementary.pdf]

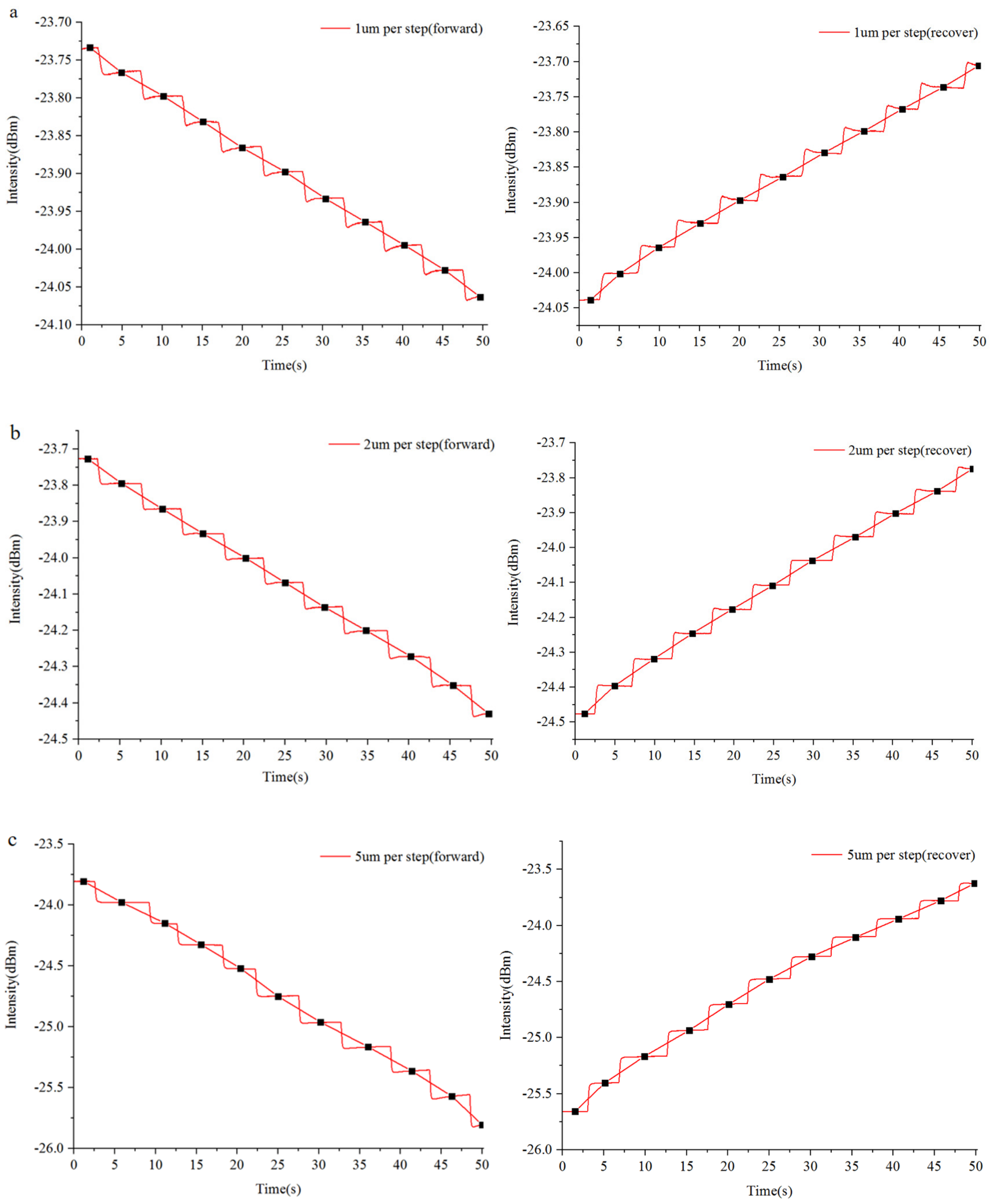

Figure S1. Characterization of 3um parameter SOFS for strain sensing. Changes of output optical intensity derived from bend and recover a SOFS. Each step corresponding to a displacement of (a) 1 um, (b) 2 um, (c) 5 um, respectively.

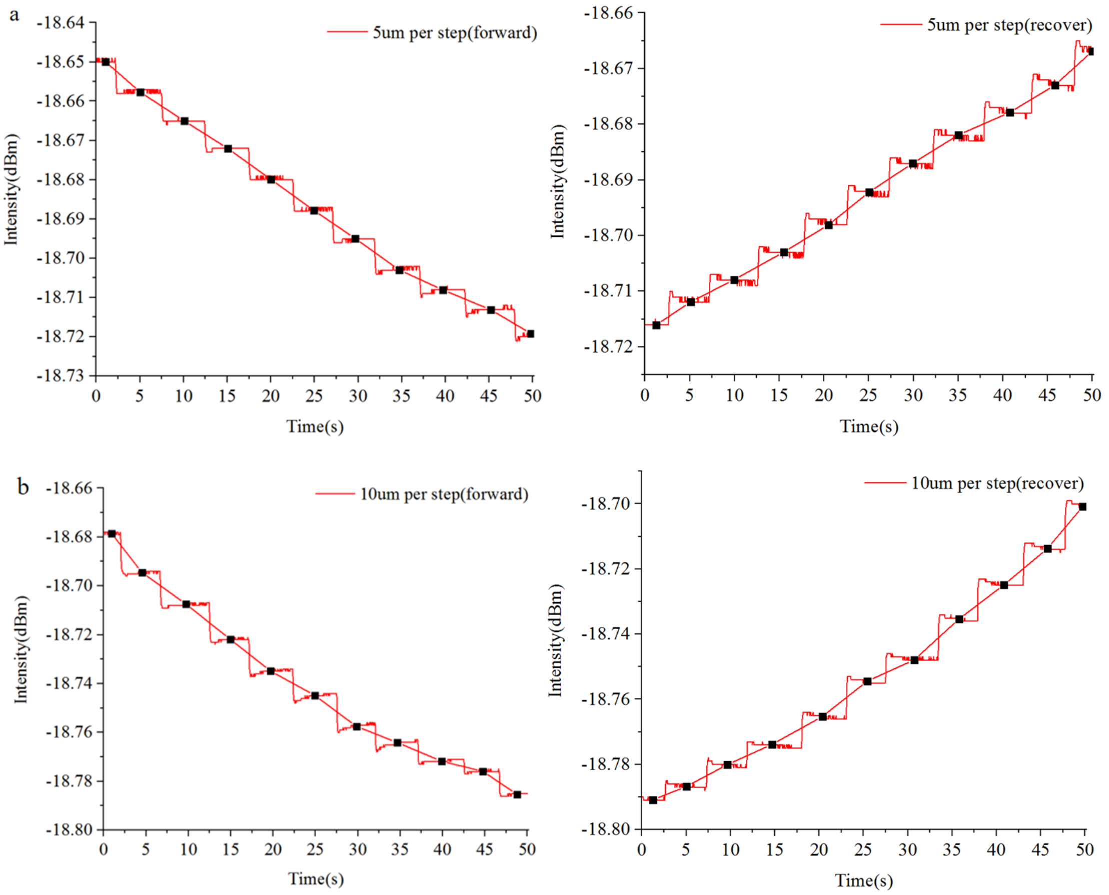

Figure S2. Characterization of 5um parameter SOFS for strain sensing. Changes of output optical intensity derived from bend and recover a SOFS. Each step corresponding to a displacement of (a) 1 um, (b) 2 um, respectively.

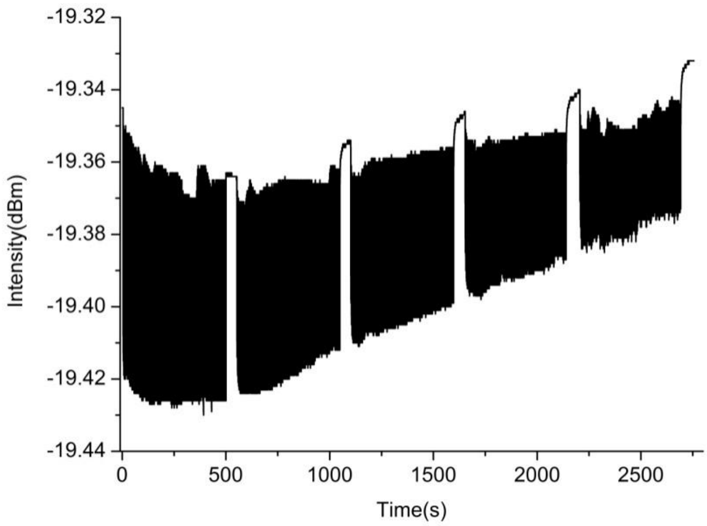

Figure S3. Measurement of the durability test under a bend of 100 um at a frequency of ~0.5 Hz for 1000 cycles in all.

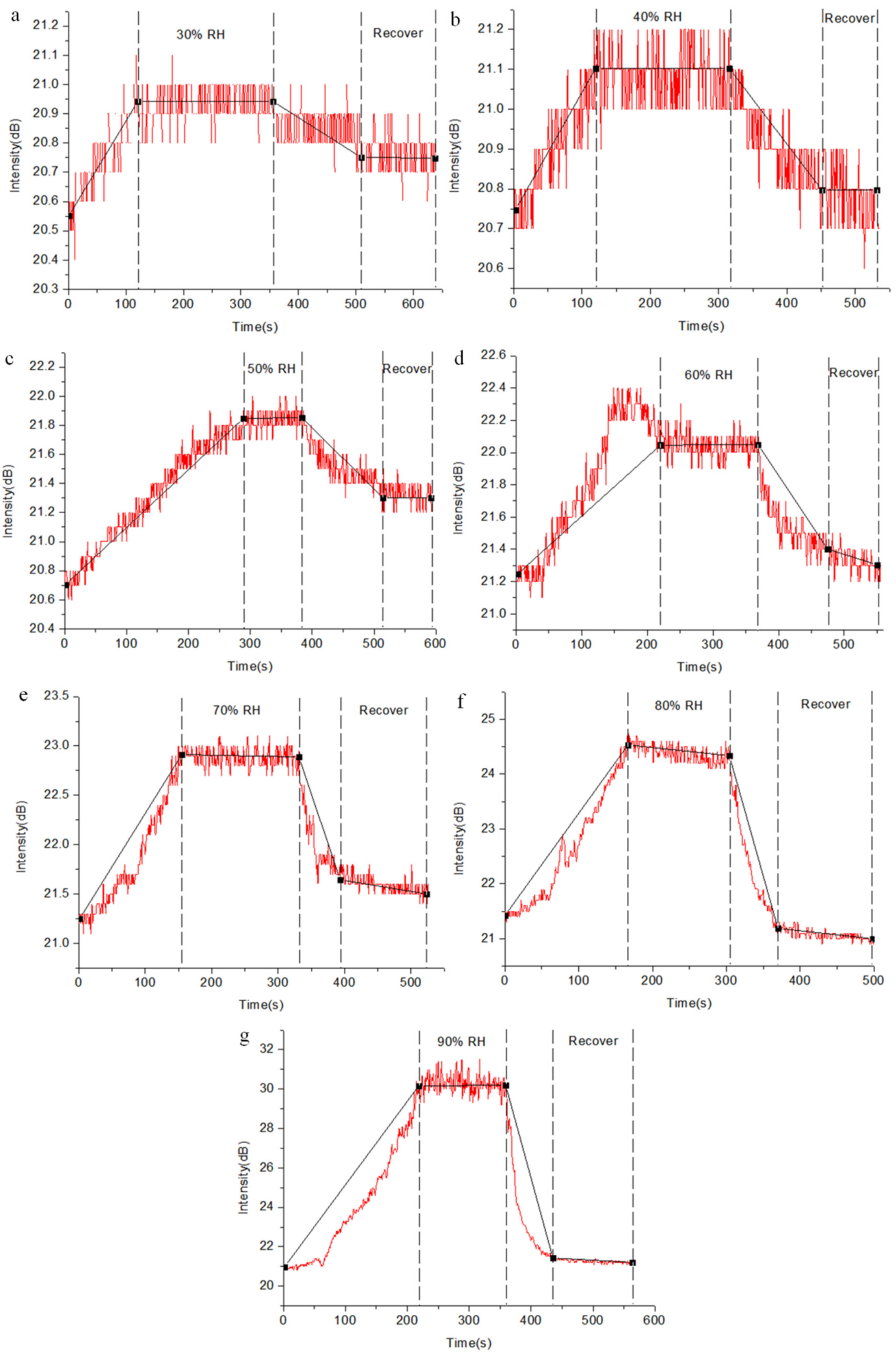

Figure S4. Humidity sensing of as-fabricated SOFS in different humidity and recover cycles, including (a) 30% RH and recover cycle, (b) 40% RH and recover cycle, (c) 50% RH and recover cycle, (d) 60% RH and recover cycle, (e) 70% RH and recover cycle, (f) 80% RH and recover cycle and (g) 90% RH and recover cycle, respectively.

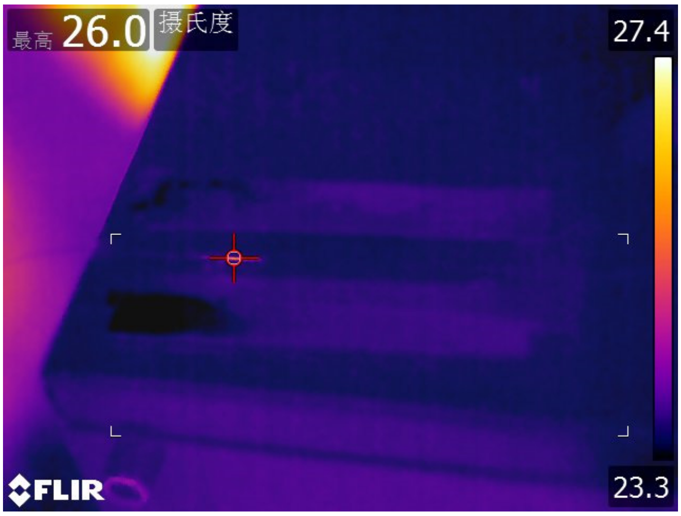

Figure S5. Infrared photograph of MNF deposited on tin oxide film, the red cross is the highest temperature area within MNF and the guided light direction is from left to right.
